# Optimized paired‐sgRNA/Cas9 cloning and expression cassette triggers high‐efficiency multiplex genome editing in kiwifruit

**DOI:** 10.1111/pbi.12884

**Published:** 2018-02-06

**Authors:** Zupeng Wang, Shuaibin Wang, Dawei Li, Qiong Zhang, Li Li, Caihong Zhong, Yifei Liu, Hongwen Huang

**Affiliations:** ^1^ Key Laboratory of Plant Resources Conservation and Sustainable Utilization South China Botanical Garden The Chinese Academy of Sciences Guangzhou Guangdong China; ^2^ Guangdong Provincial Key Laboratory of Applied Botany Guangzhou Guangdong China; ^3^ University of Chinese Academy of Sciences Beijing China; ^4^ Key Laboratory of Plant Germplasm Enhancement and Specially Agriculture Wuhan Botanical Garden The Chinese Academy of Sciences Wuhan Hubei China

**Keywords:** kiwifruit, genome editing, PTG/Cas9, CRISPR/Cas9, chromosomal fragment deletion, paired‐sgRNA

## Abstract

Kiwifruit is an important fruit crop; however, technologies for its functional genomic and molecular improvement are limited. The clustered regulatory interspaced short palindromic repeats (CRISPR)/CRISPR‐associated protein (Cas) system has been successfully applied to genetic improvement in many crops, but its editing capability is variable depending on the different combinations of the synthetic guide RNA (sgRNA) and Cas9 protein expression devices. Optimizing conditions for its use within a particular species is therefore needed to achieve highly efficient genome editing. In this study, we developed a new cloning strategy for generating paired‐sgRNA/Cas9 vectors containing four sgRNAs targeting the kiwifruit phytoene desaturase gene (*AcPDS*). Comparing to the previous method of paired‐sgRNA cloning, our strategy only requires the synthesis of two gRNA‐containing primers which largely reduces the cost. We further compared efficiencies of paired‐sgRNA/Cas9 vectors containing different sgRNA expression devices, including both the polycistronic tRNA‐sgRNA cassette (PTG) and the traditional CRISPR expression cassette. We found the mutagenesis frequency of the PTG/Cas9 system was 10‐fold higher than that of the CRISPR/Cas9 system, coinciding with the relative expressions of sgRNAs in two different expression cassettes. In particular, we identified large chromosomal fragment deletions induced by the paired‐sgRNAs of the PTG/Cas9 system. Finally, as expected, we found both systems can successfully induce the albino phenotype of kiwifruit plantlets regenerated from the G418‐resistance callus lines. We conclude that the PTG/Cas9 system is a more powerful system than the traditional CRISPR/Cas9 system for kiwifruit genome editing, which provides valuable clues for optimizing CRISPR/Cas9 editing system in other plants.

## Introduction

Creation of mutants is an important procedure in investigating gene function and crop improvement (Gaj *et al*., 2013). Compared to traditional mutagenesis, targeted genome editing mediated by site‐specific nucleases (SSNs) is precise, convenient and time‐saving (Gaj *et al*., 2013). Three SSNs, namely, zinc‐finger nucleases (ZFNs), transcription activator‐like effector nucleases (TALENs) and the clustered regulatory interspaced short palindromic repeats (CRISPR)/CRISPR‐associated protein (Cas) system (CRISPR/Cas), are widely used in targeted genome editing, particularly in crop improvement (Carroll, 2014; Chen and Gao, 2014). These SSNs induce DNA double‐strand breaks (DSBs) at specific sites and trigger *in vivo* DNA repair pathways, including homologous recombination (HR) and nonhomologous end‐joining (NHEJ) pathways (Carroll, 2014). Comparatively, ZFNs and TALENs are constructed by designing and fusing customizable ZF/TALE DNA‐binding domains with the *Fok*I cleavage domain, and their use is limited by the need to engineer a specific protein pair for each new target. The CRISPR/Cas system, which developed from the adaptive immunity of bacteria and archaea, is relatively versatile and highly efficient in targeted genome editing, in particular for multiplex engineering and fewer limitations on selections of target sites (Komor *et al*., 2016).

The CRISPR/Cas system can be divided into six types (type I–VI) (Mohanraju *et al*., 2016). The type II system, which consists of a single Cas9 protein, has been widely adapted as a simple and highly efficient targeted genome editing tool (Hayward *et al*., 2013; Shan *et al*., 2013). In this system, the mature dual CRISPR RNA (crRNA) consists of a crRNA and small *trans*‐activating CRISPR RNA (tracrRNA), which forms a functional complex with the endonuclease Cas9 (Deltcheva *et al*., 2011; Jinek *et al*., 2012). The Cas9 ribonucleoprotein complex then binds to DNA at a typical protospacer adjacent motif (PAM) sequence and a protospacer matching the crRNA. Cleavage occurs at the targeted site in relation to the PAM on both strands, which is mediated by the Cas9 endonuclease domains RuvC and HNH, introducing precise DSBs that cause targeted DNA degradation (Deltcheva *et al*., 2011).

Optimization of a single synthetic guide RNA (sgRNA) to replace the mature dual crRNA makes it easy to utilize the CRISPR/Cas9 system for genome editing in both prokaryotes and eukaryotes (Hsu *et al*., 2014; Jinek *et al*., 2012). To achieve editing at a single gene/target, both the Cas9 protein and a single sgRNA needed to be expressed in cells. In plants, Cas9 nucleases are usually fused with a nuclear localization signal (NLS) and expressed by the RNA polymerase II (Pol II) promoter (the cauliflower mosaic virus (CaMV) 35S promoter or gene‐specific promoters, such as the maize ubiquitin promoter), whereas sgRNAs are normally expressed by the RNA polymerase III (Pol III) promoter, including the commonly used *U3* and *U6* promoters (Li *et al*., 2013). The CRISPR/Cas9 system can be further applied to multiple genes/target modifications mediated by the co‐expressions of several different sgRNAs (Ma *et al*., 2015). The sgRNA expression cassette comprises a Pol III promoter, the sgRNA and a Pol III terminator, and the co‐expression of several sgRNAs can be achieved by connecting multiple sgRNA expression cassettes tandemly to simultaneously modify multiple genomic targets (Ma *et al*., 2015). However, this approach would be a challenge for most organisms due to the limitation of delivery method and plasmid vector capacity.

Recently, a scheme mediated by the endogenous tRNA‐processing system has been designed to boost the multiplex editing capability of the CRISPR/Cas9 system (Xie *et al*., 2015). In this scheme, the sgRNA expression cassette consists of a Pol III promoter, multiple tandemly arrayed tRNA‐sgRNA units and a Pol III terminator that form a polycistronic tRNA‐sgRNA (PTG) gene. The start and end sites of the tRNA in the tandemly arrayed tRNA‐sgRNA transcripts are precisely recognized and cleaved by endogenous RNases (RNase P and RNase Z in plants) to simultaneously produce multiple functional sgRNAs (Xie *et al*., 2015). Additionally, the tRNA gene contains internal promoter elements, suggesting that it may work as a potential transcriptional enhancer, as verified in a recent study involving rice, which showed that the relative expression level of the sgRNA in the PTG/Cas9 system is higher than in the CRISPR/Cas9 system (Minkenberg *et al*., 2016; Xie *et al*., 2015). Compared to the traditional CRISPR/Cas9 system, the PTG/Cas9 system is simpler and more efficient for multiplex targeted genome modifications.

The CRISPR/Cas9 system has been utilized for targeted genomic alternations in many plants, including *Arabidopsis thaliana* (Lowder *et al*., 2015), *Nicotiana benthamiana* (Li *et al*., 2013), rice (Shan *et al*., 2013), wheat (Li *et al*., 2013), maize (Feng *et al*., 2016), orange (Jia and Nian, 2014), apple (Nishitani *et al*., 2016), tomato (Pan *et al*., 2016), grape (Wang *et al*., 2017b) and *Populus* (Fan *et al*., 2015). However, the mutagenesis frequencies of the CRISPR/Cas9 system can vary significantly in plants even within the same species (e.g. 1.1%–90.4% in *Arabidopsis*) (Bortesi *et al*., 2016). The efficiency of the CRISPR/Cas9 system is influenced by many factors, especially the expression of the components in the CRISPR/Cas9 system and the nature of the sgRNA (Bortesi *et al*., 2016). For example, high expression levels of sgRNA and Cas9 can increase the editing efficiency at least in tomato (Pan *et al*., 2016), *Arabidopsis* (Ma *et al*., 2015) and rice (Xie *et al*., 2015). Moreover, sgRNAs with high GC content (greater than 50%) often have high editing efficiency (Bortesi *et al*., 2016). Optimization was therefore needed for each particular species to achieve high editing efficiency.

Kiwifruit (*Actinidia* Lindl.) is an economically and nutritionally important fruit crop that has high vitamin C content as well as minerals, dietary fibre and health‐promoting metabolites (Huang *et al*., 2013; Park *et al*., 2013). The worldwide yield of kiwifruit in 2014 reached 5 287 605 tons (http://www.fao.org/). However, the current kiwifruit industry is facing great challenges, including the outbreak of bacterial canker disease (McCann *et al*., 2017). Rapidly breeding new germplasm/cultivars with desired traits are therefore needed to solve the problems affecting the global kiwifruit industry. Because generating new kiwifruit germplasm/cultivars using conventional breeding programs is generally tedious and time‐consuming (Huang, 2014), the recently developed CRISPR/Cas9 system provides a favourable opportunity for perennial crop improvement based on its highly efficient first‐generation genome editing scheme (Shan *et al*., 2013). In particular, with the accumulation of whole‐genome sequencing data, more functional genomics studies and generation of engineered kiwifruit mutants for both breeding and production need to be conducted.

In this study, we characterized and compared the efficiencies of the CRISPR/Cas9 and the PTG/Cas9 systems in kiwifruit using *Agrobacterium*‐mediated kiwifruit transformation. We also present a rapid, efficient and inexpensive approach to generate paired‐sgRNA/Cas9 binary vectors for both the CRISPR/Cas9 and the PTG/Cas9 systems. We designed four sgRNAs (sgRNA1 to sgRNA4) that target different sites within the kiwifruit phytoene desaturase gene (*AcPDS*). We have determined that both the CRISPR/Cas9 and the PTG/Cas9 systems can be used for single and multiple site‐specific mutagenesis in kiwifruit, with efficiencies significantly higher in the PTG/Cas9 system at all four targets. The PTG/Cas9 system also induced large chromosomal fragment deletions using the paired‐sgRNA/Cas9 binary vector, and no similar mutation was found by the CRISPR/Cas9 system. Our results prove that the PTG/Cas9 system is more efficient for site‐specific mutagenesis in kiwifruit.

## Results

### Site‐specific mutagenesis strategy in kiwifruit

**Figure 1:**
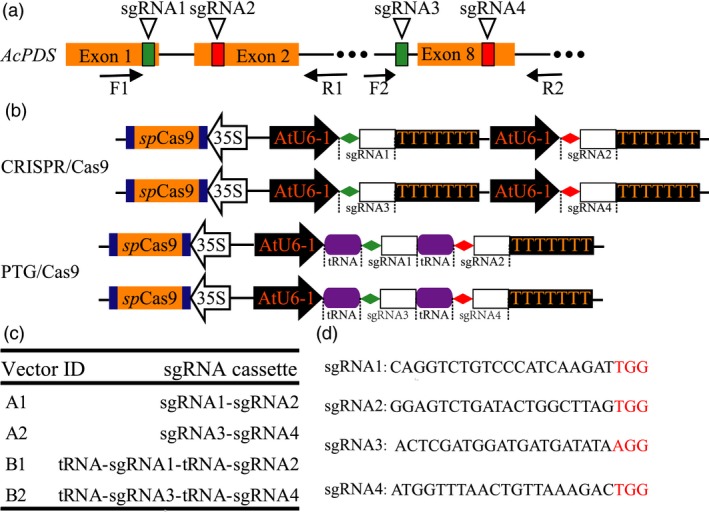
Schematic diagram of the Cas9/sgRNA binary vector and target site selection in the *AcPDS* gene. (a) Schematic illustrating the four sgRNAs targeting the *AcPDS* gene. Orange boxes indicate exons, black lines represent introns and F1/R1 and F2/R2 indicate binding sites of the primers used for PCR amplification. (b) Schematic depicting the paired‐sgRNA expression cassettes of both the CRISPR/Cas9 and the PTG/Cas9 systems. Diamonds with different colours represent sgRNA, rectangles represent the sgRNA scaffold and purple round rectangles represent tRNA. (c) The architecture of paired‐sgRNA/Cas9 binary vectors for both the CRISPR/Cas9 and the PTG/Cas9 systems. (d) The sequences of the four sgRNAs. Red letters indicate the PAM motif of each sgRNA.

The disruption of phytoene desaturase gene (*PDS*) causes albino and dwarf phenotypes in *Arabidopsis* (Qin *et al*., 2007). We identified the *PDS* homologous gene *Ach19g199631* (*AcPDS*) in kiwifruit from the kiwifruit information resource database (http://bdg.hfut.edu.cn/kir/index.html) and validated the annotation of *AcPDS* using BLASTP with the nonredundant (Nr) protein sequences database (https://www.ncbi.nlm.nih.gov/protein). A fragment of the *AcPDS* gene was amplified using the primer pair F1 and R2 (Table S1; Figure 1a) and then validated by Sanger sequencing. To design sgRNAs targeting the *AcPDS* gene, four sgRNAs (sgRNA1 to sgRNA4, Table S1) were designed to target different sites within the amplified *AcPDS* fragment using the software Cas‐Designer (Figure 1a; Park *et al*., 2015). The sgRNA1 and sgRNA2 were located on the first and second exons of the *AcPDS* gene, respectively, and both were further constructed into the same paired‐sgRNA/Cas9 binary vector (Figure 1a). The sgRNA3 and sgRNA4 were located at the intron 7 and exon 8, respectively (Figure 1a). Based on the backbone of the pYLCRISPR/Cas9P_35S_‐N vector (which were kindly provided by Prof. Yaoguang Liu, South China Agricultural University, Guangzhou, China), we inserted a fragment containing the AtU6‐1 promoter, two *Bsa*I sites, the sgRNA scaffold and the Pol III terminator to replace the sequence between the two *Asc*I sites to form a new Cas9 binary construct, which we designated as pHLW‐sgRNA‐Cas9‐AtU6‐1 (Figure S1a). Similarly, we used a fragment consisting of the AtU6‐1 promoter, tRNA^Gly^, two *Bsa*I sites, sgRNA scaffold and Pol III terminator to replace those between the two *Asc*I sites to construct another new Cas9 binary vector, pPTG‐sgRNA‐Cas9‐AtU6‐1 (Figure S1b). To construct the sgRNA intermediate vector for the PTG/Cas9 system, we used the fragment (containing the sgRNA scaffold and the tRNA^Gly^) to replace the sequence between the *Hind*III and *BamH*I sites of the construct pYLsgRNA‐AtU6‐1 (Figure S1c) to form a new sgRNA intermediate construct pHLW‐sgRNA‐tRNA (Figure S1d) (Ma *et al*., 2015).

**Figure 2:**
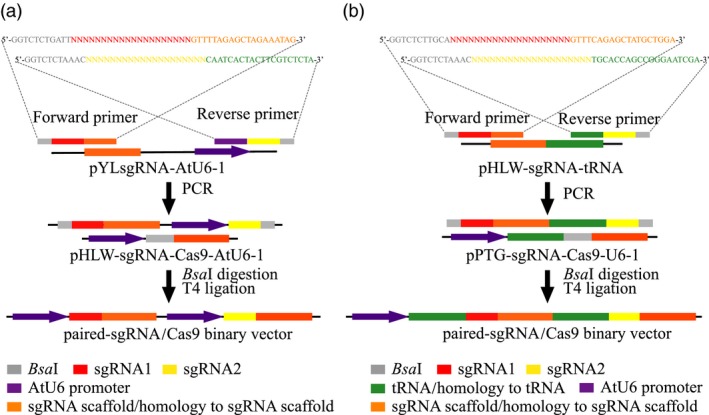
Overview of the paired‐sgRNA cloning strategy. (a) The cloning strategy of the CRISPR/Cas9 system. (b) The cloning strategy of the PTG/Cas9 system.

To compare the mutagenesis efficiencies of the CRISPR/Cas9 and the PTG/Cas9 systems in kiwifruit, we inserted paired‐sgRNA expressing cassettes of both systems into the Cas9 binary vectors (pHLW‐sgRNA‐Cas9‐AtU6‐1/pPTG‐sgRNA‐Cas9‐AtU6‐1, Figure 1b) to construct sgRNA/Cas9 binary vectors for further *Agrobacterium*‐mediated kiwifruit transformation (A1 and A2 for the CRISPR/Cas9 system; B1 and B2 for the PTG/Cas9 system; Figure 1c,d). We designed a rapid and inexpensive paired‐sgRNA cloning strategy for both the CRISPR/Cas9 and the PTG/Cas9 systems (Figure 2). This cloning strategy included two steps: the first involved routine PCR, in which primers specifically designed for both systems (Figure 2) were used to amplify sequences on the templates in relation to the sgRNA intermediate vectors pYLsgRNA‐AtU6‐1 and pHLW‐sgRNA‐tRNA for both systems, respectively (Figure 2). The second step was a cleavage and ligation reaction. Here, the PCR products and both Cas9 binary vectors (pHLW‐sgRNA‐Cas9‐AtU6‐1 for the CRISPR/Cas9 system and pPTG‐sgRNA‐Cas9‐U6‐1 for the PTG/Cas9 system) were digested with *Bsa*I, and the digestion products were then ligated with T4 DNA ligase. This generated the final paired‐sgRNA/Cas9 binary vectors (Figure 2). We used the four sgRNAs (sgRNA1 to sgRNA4) that targeted the *AcPDS* gene and tested whether this strategy can correctly and efficiently work. We successfully amplified fragments for both the CRISPR/Cas9 and the PTG/Cas9 systems using the corresponding primers (Table S1). The PCR products of both systems were of the expected lengths (508 and 227 bp for the CRISPR/Cas9 and the PTG/Cas9 systems, respectively; Figure S2). We used the primer pair SP‐DL and SP‐R to amplify the final paired‐sgRNA/Cas9 binary vectors for the CRISPR/Cas9 and PTG/Cas9 systems, followed by validation using Sanger sequencing (Table S1; Figure S2). These results revealed that the paired‐sgRNA cloning strategy can efficiently and correctly work for constructing paired‐sgRNA/Cas9 binary vectors.

### High‐efficiency multiplex editing in kiwifruit using sgRNA/Cas9 constructs

A previous study has described a highly efficient *Agrobacterium*‐mediated kiwifruit transformation system (Wang *et al*., 2010). On the basis of this system, the paired‐sgRNA/Cas9 binary vectors (A1/A2 for the CRISPR/Cas9 system and B1/B2 for the PTG/Cas9 system, Figure 1) were transferred into *Agrobacterium* strain EHA105 cells, which were further used to transfer kiwifruit leaf discs. After G418 resistance screening, the G418‐resistant callus lines were retained and further analysed to identify mutations. The genomic DNA (gDNA) of resistant callus lines was also extracted for subsequent identification. To validate the T‐DNA insertions, the specific primers SP‐DL/SP‐R of the T‐DNA region were used in PCR amplification, which revealed that the T‐DNA region was successfully and efficiently inserted into the kiwifruit genome using the CRISPR/Cas9 and the PTG/Cas9 systems, respectively (Figure S3). PCR was performed using site‐specific primers (Table S1), and the PCR products were denatured and reannealed for the subsequent T7 endonuclease I (T7E1) assay. To precisely identify the mutations in the resistant callus lines, the PCR products were purified using a HiPure gel pure DNA mini kit (Magen, Guangzhou, China) and cloned using a pClone007 simple vector kit (TsingKe, Beijing, China), followed by validation of Sanger sequencing.

**Figure 3:**
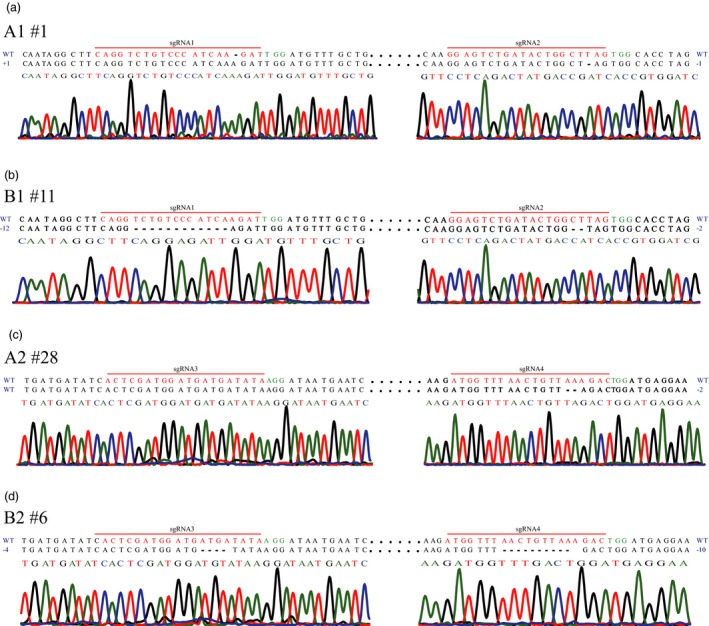
Sanger sequencing of site‐specific mutations in kiwifruit. Sequences from selected callus lines with site‐specific mutations accompanied by corresponding regions of the sequencing chromatograms are shown. The nucleotide changes (dashes for deletion and WT for wild type) are also indicated on both sides of each sequence. Dots represent nucleotides that are not shown. (a) Site‐specific editing at sgRNA1 and sgRNA2 induced by the CRISPR/Cas9 system. The sequencing chromatograms at the sgRNA2 region were the results sequenced from the reverse direction. (b) Site‐specific editing at sgRNA1 and sgRNA2 induced by the PTG/Cas9 system. The sequencing chromatograms at the sgRNA2 region were the results sequenced from the reverse direction. (c) Site‐specific editing at sgRNA3 and sgRNA4 induced by the CRISPR/Cas9 system. (d) Site‐specific editing at sgRNA3 and sgRNA4 induced by the PTG/Cas9 system.

Mutagenesis frequencies at the sgRNA1 and sgRNA2 targets in G418‐resistant callus lines transferred with vectors A1 of the CRISPR/Cas9 and B1 of the PTG/Cas9 systems were examined. For both targets, 7.50% (9 of 120) positive clones contained fragments harbouring indels amplified from callus lines transferred with vector A1, and the ratio of positive clones in relation to vector B1 reached 90.83% (109 of 120) (Table S2). And approximately 8.33% (1 of 12) of the callus lines of A1 harboured indels (Table 1; Figure 3a), whereas 91.67% (11 of 12) of the callus lines of B1 were mutated (Table 1; Figure 3b). Additionally, 8.33% (1 of 12) and 83.33% (10 of 12) of the callus lines of A1 and B1, respectively, harboured indels at both sgRNA1 and sgRNA2 (Table 1; Figure 3a,b). Similarly, mutagenesis frequencies at the sgRNA3 and sgRNA4 targets (from A2 of the CRISPR/Cas9 and B2 of the PTG/Cas9 systems) were also estimated. 6.78% (19 of 280) positive clones associated with vector A2 and 73.84% (192 of 260) positive clones related to vector B2 attained amplified fragments harbouring indels (Table S2). No mutations were detected at sgRNA3 target of the callus lines transformed with A2, and 7.14% (2 of 28) of the callus lines transformed with A2 were mutated at the sgRNA4 target (Table 1; Figure 3c). In contrast, 76.92% (20 of 26) and 65.38% (17 of 26) of the B2 callus lines harboured indels at the sgRNA3 and sgRNA4 targets, respectively (Table 1; Figure 3d). No A2 callus line was edited at both sgRNA3 and sgRNA4 simultaneously, whereas 65.38% (17 of 26) of the B2 callus lines were edited at both sgRNA3 and sgRNA4 simultaneously (Table 1; Figure 1c,d). These results indicate that the CRISPR/Cas9 and the PTG/Cas9 systems can induce mutations in kiwifruit (Table 1; Figure 3).

**Table 1:** Summary of site‐specific mutagenesis frequencies in kiwifruit

Vector ID	sgRNA	Number of callus lines analysed	Number of mutated callus lines	Mutation frequency (%)
A1	sgRNA1	12	1	8.33
	sgRNA2		1	8.33
	sgRNA1&sgRNA2		1	8.33
A2	sgRNA3	28	0	0.00
	sgRNA4		2	7.14
	sgRNA3&sgRNA4		0	0.00
B1	sgRNA1	12	11	91.67
	sgRNA2		11	91.67
	sgRNA1&sgRNA2		10	83.33
B2	sgRNA3	26	20	76.92
	sgRNA4		17	65.38
	sgRNA3&sgRNA4		17	65.38

Interestingly, the mutagenesis frequencies at all four targets of the PTG/Cas9 system were about 10‐fold higher than those of the CRISPR/Cas9 system in kiwifruit (Table 1), indicating that the PTG/Cas9 system was more effective in achieving highly efficient site‐specific editing in kiwifruit than the CRISPR/Cas9 system. Additionally, the multiplex target editing efficiency of the PTG/Cas9 system was also 10‐fold greater than that of the CRISPR/Cas9 system (Table 1). In agreement with the findings of a previous report that tRNAs may function as transcriptional enhancers for the Pol III promoter (Xie *et al*., 2015), the relative expression levels of sgRNA1 and sgRNA2 in the PTG/Cas9 system were about 38‐ and 32‐fold higher than those in the CRISPR/Cas9 system, respectively (Figure S4a). Similarly, the relative expression levels of sgRNA3 and sgRNA4 in the PTG/Cas9 system were about 17‐ and 19‐fold higher than those in the CRISPR/Cas9 system, respectively (Figure S4b). We further found that different sgRNA targets in the same system had diverse editing frequencies, whereas sgRNA targets constructed into the same vector had similar editing frequencies (Table 1).

### Chromosomal fragment deletion induced by paired‐sgRNA/Cas9 vector

**Figure 4:**
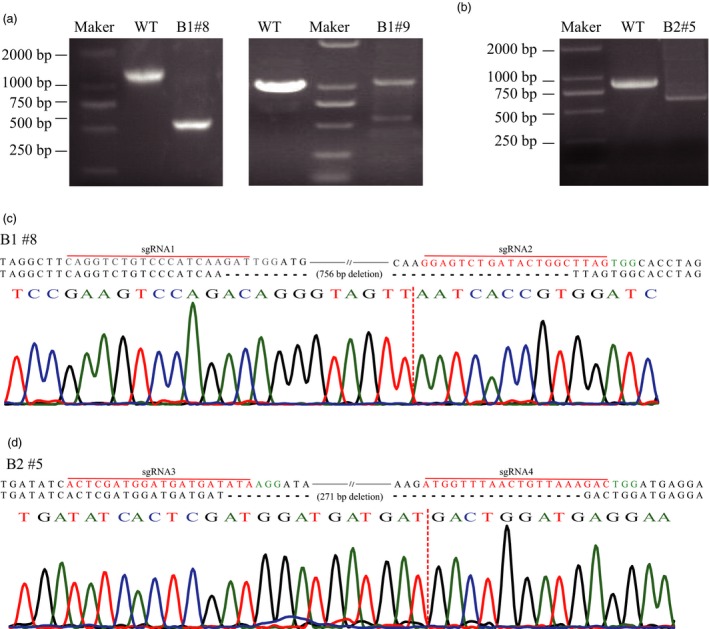
Chromosomal fragment deletion induced by paired‐sgRNA/Cas9 in kiwifruit. (a) PCR detection of chromosomal fragment deletion between sgRNA1 and sgRNA2. The truncated PCR products after agarose gel electrophoresis are shown. WT, wild type. The left gel image shows the biallelic mutant and the right is chimeric mutant. (b) PCR detection of chromosomal fragment deletion between sgRNA3 and sgRNA4. The truncated PCR products after agarose gel electrophoresis are presented. (c) to (d) Alignment of representative sequences of chromosomal fragment deletion and wild‐type sequence. The sgRNA region is labelled in red colour, and the PAM region is shown in green letters. The sequencing chromatograms of the corresponding regions are shown below the alignment. The sequencing chromatograms at the sgRNA2 region were the results sequenced from the reverse direction.

To validate the capacity of the sgRNA/Cas9 system to induce chromosomal fragment deletion between two targets (sgRNA1‐sgRNA2/sgRNA3‐sgRNA4) in kiwifruit, we used the constructs A1/A2/B1/B2 to transform kiwifruit leaf discs as previously described. We used site‐specific PCR and subsequent Sanger sequencing to precisely identify chromosomal fragment deletion. We designed site‐specific primers (F1/R1 for A1/B1 and F2/R2 for A2/B2, Table S1) for PCR amplification. We successfully identified chromosomal fragment deletion through amplification of truncated PCR products for callus lines transformed with B1 and B2 (544 bp for B1 and 630 bp for B2, Figure 4a,b). However, no chromosomal fragment deletion was detected in the CRISPR/Cas9 system (A1 and A2). Sanger sequencing further revealed that fragments between two sgRNA targets were excised from the expected sites with or without additional indels for callus lines transformed with B1 or B2 (Figure 4c,d). The length of the fragment between sgRNA1 and sgRNA2 was 755 bp, and callus line #8 transformed with B1 harboured a 755‐bp deletion that occurred at the expected sites (Figure 4c). Similarly, a 271‐bp expected fragment deletion between sgRNA3 and sgRNA4 target was identified in callus line #5, which was transformed with B2 (Figure 4d). Approximately 16.67% (2 of 12) of the callus lines transformed with B1 and 3.84% (1 of 26) of the callus lines transformed with B2 harboured the expected chromosomal fragment deletion (Figure 4). Consequently, the paired‐sgRNA/Cas9 vector of the PTG/Cas9 system can successfully induce chromosomal fragment deletion in kiwifruit (Figure 4).

### Albino phenotype induced by the sgRNA/Cas9 system in kiwifruit

**Figure 5:**
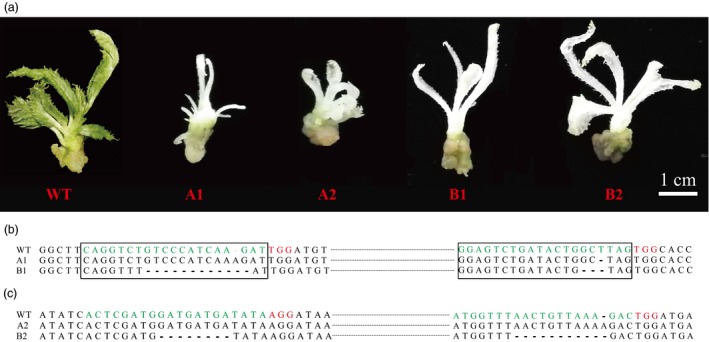
Albino phenotypes induced by site‐specific editing in kiwifruit. (a) Albino phenotypes of kiwifruit plantlets regenerated from G418‐resistant callus lines. (b) Sanger sequencing of the corresponding albino plantlets at sgRNA1 and sgRNA2 for mutation detection. (c) Sanger sequencing of corresponding albino plantlets at sgRNA3 and sgRNA4 for mutation detection.

After mutation detection using T7E1 array and Sanger sequencing, we retained mutant‐positive callus lines for subsequent regeneration. Figure 5a shows that after 4 weeks, albino leaves regenerated from mutation‐positive callus lines transformed with A1/A2/B1/B2, similar to that observed in other plants (Shan *et al*., 2013). This finding indicates that both the CRISPR/Cas9 and the PTG/Cas9 systems can successfully induce albino phenotypes in kiwifruit (Figure 5a). To precisely determine the mutation responsible for this specific phenotype, gDNA was extracted from the albino leaves, and target regions were amplified using site‐specific primers (F1/R1 for sgRNA1/sgRNA2 and F2/R2 for sgRNA3/sgRNA4, Table S1), followed by sequencing of the resulting PCR products. For the sgRNA1 and sgRNA2 targets, no mutation was identified in the wild‐type plantlets that did not express the albino phenotype, whereas a 1‐bp insertion (the sgRNA1 target) and 1‐bp deletion (the sgRNA2 target) were identified in the albino young leaves that regenerated from callus lines transformed with A1, and an 11‐bp (the sgRNA1 target) and 3‐bp deletion (the sgRNA2 target) were identified in the albino young leaves regenerated from callus lines transformed with B1 (Figure 5a,b). For the sgRNA3 and sgRNA4 targets, no mutation was identified in the wild‐type plants, whereas a 1‐bp insertion (the sgRNA4 target) was identified in the young albino leaves that regenerated from the callus lines that were transformed with A2, and 8‐bp deletions (sgRNA3 and sgRNA4 targets) were identified in the young albino leaves regenerated from callus lines that were transformed with B2 (Figure 5a,c). Our results revealed that the albino phenotype in kiwifruit can be precisely induced by site‐specific genome editing using both the CRISPR/Cas9 and the PTG/Cas9 systems.

#### Off‐target analysis

To perform the off‐target analysis, we used the online tool Cas‐OFFinder (http://www.rgenome.net/cas-offinder/) to predict the potential off‐target sites of four sgRNAs, leading to four putative off‐target sites that were identified for further analysis (Table 2). All PCR products amplified from gDNA of all kiwifruit G418‐resistant callus lines using specific primers (OT1‐4, Table S1) were cloned and sequenced. No mutation was found in any putative off‐target sites in all callus lines selected (Table 2).

**Table 2:** Off‐target analysis of kiwifruit callus lines

Target	Off‐target sites	Putative off‐target sequences	Putative off‐target loci	Number of examined lines	Number of lines with off‐targets
gRNA1	1	tAGGTCTtTCCCATCAAGAT	Chr23: ‐318123	78	0
	2	ACTCGATGGAcGAaGATATA	Chr23: ‐307000	78	0
gRNA3	3	ATGGgTTAACTGTTAAAGAC	Chr23: ‐306730	78	0
gRNA4	4	ATGGTTTAACTGTTtgAGAC	Chr28: ‐392519	78	0

## Discussion

Recently, the CRISPR/Cas system has been successfully applied in site‐specific genome editing in diverse taxa in both prokaryotes and eukaryotes (Doench *et al*., 2016; Dow *et al*., 2015; Kleinstiver *et al*., 2016; Makarova *et al*., 2011; Shan *et al*., 2013). This system had been modified to precisely control gene transcription, including transcriptional activation and repression (Chavez *et al*., 2015; Kiani *et al*., 2014, 2015), and it is considered a robust and versatile toolkit for functional genomic research, including crop improvement and molecular breeding in plants (Komor *et al*., 2016). Our application of the CRISPR/Cas9 system in the genus *Actinidia* will broaden its applications to the plant kingdom and improve functional genomic research and germplasm enhancement in *Actinidia*.

A previous research described a method for paired‐sgRNA cloning (Vidigal and Ventura, 2015). This strategy requires the synthesis of a 110‐bp DNA oligo and two primers (Vidigal and Ventura, 2015). In the present study, we developed a new strategy for paired‐sgRNA cloning, which just requires the synthesis of two sgRNA‐containing primers (Figure 2). Comparatively, the cost of synthesizing the 110‐bp DNA oligo was about sevenfold higher than that of synthesizing two sgRNA‐containing primers. Moreover, our strategy did not include the step of Gibson ligation and only comprised two steps of PCR and ligation (Figure 2), resulting into a time‐saving process of cloning sgRNA pairs. Although the earlier method mentioned was more suitable for high‐throughput vector construction on the basis of a pair of universal primers, our strategy with two sgRNA‐containing primers was simpler and cheaper which are more useful for paired‐sgRNA cloning in site‐specific genomic editing (Figure S2).

To evaluate the performance of the CRISPR/Cas system in kiwifruit, we compared the editing efficiencies of both the CRISPR/Cas9 and the PTG/Cas9 systems. We observed that the PTG/Cas9 system offers a 10‐fold higher target‐editing efficiency than that of the CRISPR/Cas9 system (Table 1). Our results were also in agreement with the findings of the previous report, in which the PTG/Cas9 system was shown to be more efficient than the CRISPR/Cas9 system in rice (Xie *et al*., 2015). The relatively high efficiency of the PTG/Cas9 system may be attributable to the transcriptional enhancer in the tRNA sequence (Xie *et al*., 2015). In rice, the relative expression of sgRNAs in the PTG/Cas9 system was higher than that in the CRISPR/Cas9 system, which indicates that transcriptional enhancement is induced by tRNA (Xie *et al*., 2015). In our study, we estimated the relative expression of sgRNAs in both the CRISPR/Cas9 and the PTG/Cas9 systems, which revealed that the expression level of all four sgRNAs in the PTG/Cas9 system was higher than those in the CRISPR/Cas9 system, further supporting the hypothesis that tRNA may function as a transcriptional enhancer of the Pol III promoter (Figure S4).

For multiplex target editing in kiwifruit, the efficiency of the PTG/Cas9 system was also higher than that of the CRISPR/Cas9 system (Table 1). Moreover, chromosomal fragment deletions induced by paired‐sgRNA/Cas9 vectors of the PTG/Cas9 system were detected (Figure 4). No chromosomal fragment deletion was induced by the paired‐sgRNA/Cas9 vectors of the CRISPR/Cas9 system, which may have been caused by the inefficient site‐specific editing at each target (Table 1). A previous study showed that fragment deletion efficiency is negatively correlated with the distance between two paired cut sites (Xie *et al*., 2015); however, our results were contrary to this finding (Figure 4), in which a closer distance between two paired sites showing a relatively higher fragment deletion efficiency, suggesting a positive relationship. This discordance may be attributable to the context of the sgRNAs and neighbouring genomic sequence (Doench *et al*., 2016), leading to the likelihood of species‐dependent outcomes. The different DNA cleavage efficiency at both target sites is also possibly related it. In grape, variation in mutation frequency among four targets seems to result in different frequency of large deletions between them (Wang *et al*., 2017b). Accumulating more data is definitely needed to identify the key factors determining the efficiency of large fragment deletion.

Historically, the kiwifruit breeding is mainly limited by the direct or indirect selections from natural populations in the wild, which was resource‐ and time‐consuming (Liu *et al*., 2010). The utilizations of genomic data for kiwifruit germplasm creation or cultivar improvement, however, are less carried out. In crops, the CRISPR/Cas9 system has become into a powerful tool for crop improvement and molecular breeding, such as the simultaneous editing of three homoeoalleles in hexaploid bread wheat confers broad‐spectrum resistance to powdery mildew (Wang *et al*., 2014), and a recent study engineering quantitative trait variation by genome editing in tomato (Rodríguez‐Leal *et al*., 2017). In rice, the CRISPR/Cas9‐based screening of mutant library is greatly accelerating its research and breeding (Lu *et al*., 2017; Wang *et al*., 2017a). The highly efficient, robust and precise genome editing capacity of the CRISPR/Cas9 system presented here therefore encourages us to broaden its application to genomic research and molecular breeding in kiwifruit, including systematically identifying both functional genes and mutant phenotypes in future.

## Experimental procedures

### Construct modification for rapid paired‐sgRNA cloning

For the rapid paired‐sgRNA cloning strategy, the sgRNA intermediate construct and Cas9 binary construct were modified. For the CRISPR/Cas9 system, the sgRNA intermediate construct was the vector pYLsgRNA‐AtU6‐1 (Ma *et al*., 2015), and the Cas9 binary construct was modified as follows. Fragment 1 containing the AtU6‐1 promoter and two *Bsa*I sites was amplified from vector pYLsgRNA‐AtU6‐1 using the primers U6‐1‐F and U6‐1‐R‐C (Table S1), and Fragment 2 containing two *Bsa*I sites, the sgRNA scaffold and the Pol III terminator was amplified from vector pYLsgRNA‐AtU6‐1 using primers GF and GR (Table S1). Fragment 1, fragment 2 and pYLCRISPR/Cas9P_35S_‐N linearized with *Asc*I were fused to form the Cas9 binary vector pHLW‐sgRNA‐Cas9‐AtU6‐1 using a pEASY‐Uniseamless cloning and assembly kit (Transgen, Beijing, China). For the PTG/Cas9 system, the sgRNA intermediate vector was pHLW‐sgRNA‐tRNA, and the Cas9 binary vector was pPTG‐sgRNA‐Cas9‐AtU6‐1, which were modified based on vectors pYLsgRNA‐AtU6‐1 and pYLCRISPR/Cas9P_35S_‐N, respectively. For vector pHLW‐sgRNA‐tRNA, a 177‐bp DNA oligo containing a *BamH*I site, the sgRNA scaffold, tRNA^Gly^ and a *Hind*III site was synthesized and inserted into vector pYLsgRNA‐AtU6‐1 that was linearized with *BamH*I and *Hind*III. Fragment 3 containing the AtU6‐1 promoter was amplified from the vector pYLsgRNA‐AtU6‐1 using primers U6‐1‐F and U6‐1‐R (Table S1). Fragment 4 containing the tRNA^Gly^ and a *Bsa*I site was amplified from the vector pHLW‐sgRNA‐tRNA using primers TF and TR (Table S1). Fragment 5, which contained two *Bsa*I sites, the sgRNA scaffold and the Pol III terminator, was amplified from the vector pYLsgRNA‐AtU6‐1 using primers GF and GR (Table S1). Fragments 3, 4 and 5 and pYLCRISPR/Cas9P_35S_‐N linearized with *Asc*I were fused to form the Cas9 binary vector pPTG‐sgRNA‐Cas9‐AtU6‐1 using a pEASY‐Uniseamless cloning and assembly kit (Transgen, Beijing, China).

### Paired‐sgRNA cloning and paired‐sgRNA/Cas9 expression vector construction

To test the efficiency of the CRISPR/Cas system in kiwifruit, we designed four sgRNAs targeting the *AcPDS* gene, namely, sgRNA1 to sgRNA4. Every two targets were cloned into a single Cas9 binary vector to construct paired‐sgRNA/Cas9 binary vectors. The fragment containing the first *Bsa*I site, sgRNA1, the AtU6‐1 promoter, sgRNA2 and the second *Bsa*I site was amplified from pYLsgRNA‐AtU6‐1 using primers crispr‐sgRNA1‐F and crispr‐sgRNA2‐R and inserted into vector pHLW‐sgRNA‐Cas9‐AtU6‐1 that was linearized with *Bsa*I to construct vector A1 of the CRISPR/Cas9 system (Table S1; Figure 1b). Similarly, vector A2 containing sgRNA3 and sgRNA4 of the CRSIPR/Cas9 system was constructed using primers crispr‐sgRNA3‐F and crispr‐sgRNA4‐R (Table S1; Figure 1b). The fragment containing the first *Bsa*I site, sgRNA1, tRNA^Gly^, sgRNA2 and the second *Bsa*I site was amplified from pHLW‐sgRNA‐tRNA using primers ptg‐sgRNA1‐F and ptg‐sgRNA2‐R and inserted into vector pPTG‐sgRNA‐Cas9‐AtU6‐1 that was linearized with *Bsa*I to construct vector B1 of the PTG/Cas9 system (Table S1; Figure 1b). Similarly, vector B2 containing sgRNA3 and sgRNA4 of the PTG/Cas9 system was constructed using primers ptg‐sgRNA3‐F and ptg‐sgRNA4‐R (Table S1; Figure 1b).

### Kiwifruit tissue culture and *Agrobacterium*‐mediated transformation

Kiwifruit tissue culture was performed as previously described (Akba and Namli, 2009; Yuan, 2011). The leaf discs of kiwifruit cultivar *Hongyang* were used for tissue culture and *Agrobacterium*‐mediated transformation. The high‐efficiency transformation platform for kiwifruit was also previously constructed (Wang *et al*., 2010). Plantlets regenerated from calli under G418 screening were grown in a tissue culture room with 12‐h light/12‐h dark conditions at 26 °C.

### Mutation detection

Genomic DNA was extracted from kiwifruit G418‐resistant callus lines and regenerated plants using the cetyltrimethyl ammonium bromide (CTAB) method (Murray and Thompson, 1980). To validate T‐DNA insertions, specific primers SP‐DL/SP‐R of the T‐DNA region were used in PCR amplification (Ma *et al*., 2015). The target‐specific primers (F1/R1 for sgRNA1/2 and F2/R2 for sgRNA3/4; Figure 1a) were used in the amplification of fragments containing the sgRNA targets. The PCR products were cloned into the pClone007 vector using a pClone007 simple vector kit (TsingKe, Beijing, China). The ligated products were transformed into *Escherichia coli* strain DH5α cells, and 10 positive clones were selected for Sanger sequencing. The DNAMAN software (version 4.0; Lynnon Corporation, Canada) was used for alignment analysis. The mutation efficiencies were examined as a ratio of callus lines with mutation. The fragments encompassing each target were separately amplified using the appropriate primers (Table S1). About 500 ng of the respective PCR product was used for the T7E1 array using T7 endonuclease I (NEB, Ipswich) according to the manufacturer's instruction. After the T7E1 array, the reaction products were analysed by 1.5% agarose gel electrophoresis.

### Off‐target analysis

All kiwifruit G418‐resistant callus lines used for mutation detection were also used for off‐target analysis. The potential off‐target sites of four sgRNAs were predicted using the online tool Cas‐OFFinder (http://www.rgenome.net/cas-offinder/), and four potential off‐target sites were retained (Table 2). Specific primers were designed and used for the further off‐target analysis (Table S1). The amplified products were cloned, and 10 positive clones were selected for Sanger sequencing. The alignment analysis was also performed using the DNAMAN software.

### RNA extraction, reverse transcription PCR and quantitative PCR

To validate the relative expression of four sgRNAs of both the CRISPR/Cas9 and the PTG/Cas9 systems in kiwifruit, we designed four pair primers (sgRNA1‐F/sgRNA‐R, sgRNA2‐F/sgRNA‐R, sgRNA3‐F/sgRNA‐R and sgRNA4‐F/sgRNA‐R) using the online software Primer3Plus, which were then synthesized commercially (Sangon Biotech Co., Ltd., Shanghai, China) (Table S1). RNA was extracted from callus lines transformed with A1/A2/B1/B2 using HiPure plant RNA kits (Magen, Guangzhou, China). The cDNA was prepared from all samples using a one‐step gDNA removal and cDNA Synthesis SuperMix Kit (TransGen, Beijing, China) and was then used as the input for quantitative PCR (qPCR) experiments. Each qPCR was performed in a total volume of 20 μL, containing 10 μL of Tip Green qPCR SuperMix (TransGen, Beijing, China), 0.2 μm of each primer, 1 μL of 1 : 5 diluted cDNA and 8.2 μL ddH_2_O. Thermal cycling consisted of a hold at 94 °C for 30 s, followed by 40 cycles of 94 °C for 5 s and 60 °C for 30 s. The temperature was then gradually raised by 0.5 °C every 10 s for performing melting curve analysis. Each sample was amplified in triplicate, and all qPCRs were performed on a LightCycler 480 system (Roche, Basel, Switzerland). The ΔΔCt method was employed with *Achn107181* (kiwifruit actin gene) and *Achn381211* (protein phosphatase 2A, PP2A‐like gene) as endogenous controls (Petriccione *et al*., 2015).

## Conflict of interest

The authors declare that they have no competing interests.

## Author contributions

HH and YL directed the study. ZW and YL designed the experiments. LL, DL and CZ contributed to sample and tissue collection. ZW and SW performed the tissue culture and mutation identification. ZW, YL, QZ and SW performed the data processing. ZW performed the quantitative RT‐PCR experiments. ZW and YL drafted the manuscript. All authors approved the final draft.

## Supporting information


**Figure S1** Overall structures of vectors used in the CRISPR/Cas or PTG/Cas9 systems.
**Figure S2** PCR validation and Sanger sequencing of PCR products amplified from paired‐sgRNA/Cas9 vectors.
**Figure S3** Validation of transgene‐positive cells in G418‐resistant callus lines using Cas9‐specific primers SP‐DL/SP‐R.
**Figure S4** Relative expression of sgRNAs in kiwifruit.
**Table S1** Primers used for vectors construction, mutation detection, off‐target analysis and qPCR.
**Table S2** Summary of mutagenesis frequencies in positive clones selected.

## References

[pbi12884-bib-0001] Akba, F. and Namli, S. (2009) Callus induction and plant regeneration from different explants of *Actinidia deliciosa* . Appl. Biochem. Biotechnol. 158, 470–475. 18975140 10.1007/s12010-008-8401-2

[pbi12884-bib-0002] Bortesi, L. , Zhu, C. , Zischewski, J. , Perez, L. , Bassié, L. , Nadi, R. , Forni, G. *et al* (2016) Patterns of CRISPR/Cas9 activity in plants, animals and microbes. Plant Biotechnol. J. 14, 2203–2216. 27614091 10.1111/pbi.12634 PMC5103219

[pbi12884-bib-0003] Carroll, D. (2014) Genome engineering with targetable nucleases. Annu. Rev. Biochem. 83, 409–439. 24606144 10.1146/annurev-biochem-060713-035418

[pbi12884-bib-0004] Chavez, A. , Scheiman, J. , Vora, S. , Pruitt, B.W. , Tuttle, M. , Iyer, E.P. , Lin, S. *et al* (2015) Highly efficient Cas9‐mediated transcriptional programming. Nat. Methods, 12, 326–328. 25730490 10.1038/nmeth.3312 PMC4393883

[pbi12884-bib-0005] Chen, K. and Gao, C. (2014) Targeted genome modification technologies and their applications in crop improvements. Plant Cell Rep. 33, 575–583. 24277082 10.1007/s00299-013-1539-6

[pbi12884-bib-0006] Deltcheva, E. , Chylinski, K. , Sharma, C.M. , Gonzales, K. , Chao, Y. , Pirzada, Z.A. , Eckert, M.R. *et al* (2011) CRISPR RNA maturation by *trans*‐encoded small RNA and host factor RNase III. Nature, 471, 602–607. 21455174 10.1038/nature09886 PMC3070239

[pbi12884-bib-0007] Doench, J.G. , Fusi, N. , Sullender, M. , Hegde, M. , Vaimberg, E.W. , Donovan, K.F. *et al* (2016) Optimized sgRNA design to maximize activity and minimize off‐target effects of CRISPR‐Cas9. Nat. Biotechnol. 33, 390–394. 10.1038/nbt.3437 PMC4744125 26780180

[pbi12884-bib-0008] Dow, L.E. , Fisher, J. , O'Rourke, K.P. , Muley, A. , Kastenhuber, E.R. , Livshits, G. *et al* (2015) Inducible in vivo genome editing with CRISPR‐Cas9. Nat. Biotechnol. 33, 390–394. 25690852 10.1038/nbt.3155 PMC4390466

[pbi12884-bib-0009] Fan, D. , Liu, T. , Li, C. , Jiao, B. , Li, S. , Hou, Y. and Luo, K. (2015) Efficient CRISPR/Cas9‐mediated targeted mutagenesis in *Populus* in the first generation. Sci. Rep. 5, 12217. 26193631 10.1038/srep12217 PMC4507398

[pbi12884-bib-0010] Feng, C. , Yuan, J. , Wang, R. , Liu, Y. , Birchler, J.A. and Han, F. (2016) Efficient targeted genome modification in maize using CRISPR/Cas9 system. J. Genet. Genomics, 43, 37–43. 26842992 10.1016/j.jgg.2015.10.002

[pbi12884-bib-0011] Gaj, T. , Gersbach, C.A. and Barbas, C.F. (2013) ZFN, TALEN, and CRISPR/Cas‐based methods for genome engineering. Trends Biotechnol. 31, 397–405. 23664777 10.1016/j.tibtech.2013.04.004 PMC3694601

[pbi12884-bib-0012] Hayward, V. , Tatsuta, M. , Yano, H. , Iishi, H. , Ishiguro, S. , Maeda, S. *et al* (2013) Multiplex genome engineering using CRISPR/Cas systems. Science, 339, 1504–1508.

[pbi12884-bib-0013] Hsu, P.D. , Lander, E.S. and Zhang, F. (2014) Development and applications of CRISPR‐Cas9 for genome engineering. Cell, 157, 1262–1278. 24906146 10.1016/j.cell.2014.05.010 PMC4343198

[pbi12884-bib-0014] Huang, H. (2014) The Genus Actinidia, a World Monograph. Beijing, China: Science Press.

[pbi12884-bib-0015] Huang, S. , Ding, J. , Deng, D. , Tang, W. , Sun, H. , Liu, D. , Zhang, L. *et al* (2013) Draft genome of the kiwifruit *Actinidia chinensis* . Nat. Commun. 4, 1–9. 10.1038/ncomms3640 PMC4089393 24136039

[pbi12884-bib-0016] Jia, H. and Nian, W. (2014) Targeted genome editing of sweet orange using Cas9/sgRNA. PLoS ONE, 9, e93806. 24710347 10.1371/journal.pone.0093806 PMC3977896

[pbi12884-bib-0017] Jinek, M. , Chylinski, K. , Fonfara, I. , Hauer, M. , Doudna, J.A. and Charpentier, E. (2012) A programmable dual‐RNA–guided DNA endonuclease in adaptive bacterial immunity. Science, 337, 816–822. 22745249 10.1126/science.1225829 PMC6286148

[pbi12884-bib-0018] Kiani, S. , Beal, J. , Ebrahimkhani, M.R. , Huh, J. , Hall, R.N. , Xie, Z. , Li, Y. *et al* (2014) CRISPR transcriptional repression devices and layered circuits in mammalian cells. Nat. Methods, 11, 723–726. 24797424 10.1038/nmeth.2969 PMC4228775

[pbi12884-bib-0019] Kiani, S. , Chavez, A. , Tuttle, M. , Hall, R.N. , Chari, R. , Ter‐Ovanesyan, D. , Qian, J. *et al* (2015) Cas9 sgRNA engineering for genome editing, activation and repression. Nat. Methods, 12, 1051–1054. 26344044 10.1038/nmeth.3580 PMC4666719

[pbi12884-bib-0020] Kleinstiver, B.P. , Pattanayak, V. , Prew, M.S. , Tsai, S.Q. , Nguyen, N.T. , Zheng, Z. and Joung, J.K. (2016) High‐fidelity CRISPR–Cas9 nucleases with no detectable genome‐wide off‐target effects. Nature, 529, 490–495. 26735016 10.1038/nature16526 PMC4851738

[pbi12884-bib-0021] Komor, A.C. , Badran, A.H. and Liu, D.R. (2016) CRISPR‐based technologies for the manipulation of eukaryotic genomes. Cell, 168, 1–17. 10.1016/j.cell.2016.10.044 PMC5235943 27866654

[pbi12884-bib-0022] Li, J.F. , Norville, J.E. , Aach, J. , McCormack, M. , Zhang, D. , Bush, J. , Church, G.M. *et al* (2013) Multiplex and homologous recombination–mediated genome editing in *Arabidopsis* and *Nicotiana benthamiana* using guide RNA and Cas9. Nat. Biotechnol. 31, 688–691. 23929339 10.1038/nbt.2654 PMC4078740

[pbi12884-bib-0023] Liu, Y. , Liu, Y. and Huang, H. (2010) Genetic variation and natural hybridization among sympatric *Actinidia* species and the implications for introgression breeding of kiwifruit. Tree Genet. Genomes, 6, 801–813.

[pbi12884-bib-0024] Lowder, L.G. , Zhang, D. , Baltes, N.J. , Paul, J.W. , Tang, X. , Zheng, X. , Voytas, D.F. *et al* (2015) A CRISPR/Cas9 toolbox for multiplexed plant genome editing and transcriptional regulation. Plant. Pathol. 169, 971–985. 10.1104/pp.15.00636 PMC4587453 26297141

[pbi12884-bib-0025] Lu, Y. , Ye, X. , Guo, R. , Huang, J. , Wang, W. , Tang, J. , Tan, L. *et al* (2017) Genome‐wide targeted mutagenesis in rice using the CRISPR/Cas9 system. Mol Plant. 10, 1242–1245. 28645638 10.1016/j.molp.2017.06.007

[pbi12884-bib-0026] Ma, X. , Zhang, Q. , Zhu, Q. , Liu, W. , Chen, Y. , Qiu, R. , Wang, B. *et al* (2015) A robust CRISPR/Cas9 system for convenient, high‐efficiency multiplex genome editing in monocot and dicot plants. Mol Plant. 8, 1274–1284. 25917172 10.1016/j.molp.2015.04.007

[pbi12884-bib-0027] Makarova, K.S. , Haft, D.H. , Barrangou, R. , Brouns, S.J.J. , Charpentier, E. , Horvath, P. , Moineau, S. *et al* (2011) Evolution and classification of the CRISPR–Cas systems. Nat. Rev. Microbiol. 9, 467–477. 21552286 10.1038/nrmicro2577 PMC3380444

[pbi12884-bib-0028] McCann, H.C. , Li, L. , Liu, Y. , Li, D. , Pan, H. , Zhong, C. , Rikkerink, E.H. *et al* (2017) Origin and evolution of the kiwifruit canker pandemic. Genome Biol. Evol. 9, 932–944. 28369338 10.1093/gbe/evx055 PMC5388287

[pbi12884-bib-0029] Minkenberg, B. , Xie, K. and Yang, Y. (2016) Discovery of rice essential genes by characterizing CRISPR‐edited mutation of closely related rice MAP kinase genes. Plant J. 89, 636–648. 10.1111/tpj.13399 27747971

[pbi12884-bib-0030] Mohanraju, P. , Makarova, K.S. , Zetsche, B. , Zhang, F. , Koonin, E.V. and Van der Oost, J. (2016) Diverse evolutionary roots and mechanistic variations of the CRISPR‐Cas systems. Science, 353, aad5147. 27493190 10.1126/science.aad5147 PMC13189112

[pbi12884-bib-0031] Murray, M.G. and Thompson, W.F. (1980) Rapid isolation of high molecular weight plant DNA. Nucleic Acids Res. 8, 4321–4326. 7433111 10.1093/nar/8.19.4321 PMC324241

[pbi12884-bib-0032] Nishitani, C. , Hirai, N. , Komori, S. , Wada, M. , Okada, K. , Osakabe, K. , Yamamoto, T. *et al* (2016) Efficient genome editing in apple using a CRISPR/Cas9 system. Sci. Rep. 6, 31481. 27530958 10.1038/srep31481 PMC4987624

[pbi12884-bib-0033] Pan, C. , Ye, L. , Qin, L. , Liu, X. , He, Y. , Wang, J. , Chen, L. *et al* (2016) CRISPR/Cas9‐mediated efficient and heritable targeted mutagenesis in tomato plants in the first and later generations. Sci. Rep. 6, 24765. 27097775 10.1038/srep24765 PMC4838866

[pbi12884-bib-0034] Park, Y.S. , Im, M.H. , Ham, K.S. , Kang, S.G. , Park, Y.K. , Namiesnik, J. , Leontowicz, H. *et al* (2013) Nutritional and pharmaceutical properties of bioactive compounds in organic and conventional growing Kiwifruit. Plant Foods Hum. Nutr. 68, 57–64. 23386202 10.1007/s11130-013-0339-z

[pbi12884-bib-0035] Park, J. , Bae, S. and Kim, J. (2015) Sequence analysis Cas‐Designer: a web‐based tool for choice of CRISPR‐Cas9 target sites. Bioinformatics, 31, 4014–4016. 26358729 10.1093/bioinformatics/btv537

[pbi12884-bib-0036] Petriccione, M. , Mastrobuoni, F. , Zampella, L. and Scortichini, M. (2015) Reference gene selection for normalization of RT‐qPCR gene expression data from *Actinidia deliciosa* leaves infected with *Pseudomonas syringae* pv. *actinidiae* . Sci. Rep. 5, 16961. 26581656 10.1038/srep16961 PMC4652207

[pbi12884-bib-0037] Qin, G. , Gu, H. , Ma, L. , Peng, Y. , Deng, X.W. , Chen, Z. and Qu, L.J. (2007) Disruption of phytoene desaturase gene results in albino and dwarf phenotypes in *Arabidopsis* by impairing chlorophyll, carotenoid, and gibberellin biosynthesis. Cell Res. 17, 471–482. 17486124 10.1038/cr.2007.40

[pbi12884-bib-0038] Rodríguez‐Leal, D. , Lemmon, Z.H. , Man, J. , Bartlett, M.E. and Lippman, Z.B. (2017) Engineering quantitative trait variation for crop improvement by genome editing. Cell, 171, 470–480. 28919077 10.1016/j.cell.2017.08.030

[pbi12884-bib-0039] Shan, Q. , Wang, Y. , Li, J. , Zhang, Y. , Chen, K. , Liang, Z. , Zhang, K. *et al* (2013) Targeted genome modification of crop plants using a CRISPR‐Cas system. Nat. Biotechnol. 31, 684–686. 23929338 10.1038/nbt.2650

[pbi12884-bib-0040] Vidigal, J.A. and Ventura, A. (2015) Rapid and efficient one‐step generation of paired sgRNA CRISPR‐Cas9 libraries. Nat. Commun. 6, 1–7. 10.1038/ncomms9083 PMC4544769 26278926

[pbi12884-bib-0041] Wang, T. , Karunairetnam, S. , Wu, R. , Wang, Y.Y. and Gleave, A.P. (2010) High efficiency transformation platforms for kiwifruit (*Actinidia* spp.) functional genomics In XXVIII International Horticultural Congress on Science and Horticulture for People (IHC2010): International Symposium on 929, pp. 143–148.

[pbi12884-bib-0042] Wang, Y. , Cheng, X. , Shan, Q. , Zhang, Y. , Liu, J. , Gao, C. and Qiu, J.L. (2014) Simultaneous editing of three homoeoalleles in hexaploid bread wheat confers heritable resistance to powdery mildew. Nat. Biotechnol. 32, 947–952. 25038773 10.1038/nbt.2969

[pbi12884-bib-0043] Wang, L. , Zheng, J. , Luo, Y. , Xu, T. , Zhang, Q. , Zhang, L. *et al* (2017a) Construction of a genome‐wide mutant library in rice using CRISPR/Cas9. Mol Plant. 10, 1238–1241. 28645639 10.1016/j.molp.2017.06.006

[pbi12884-bib-0044] Wang, X. , Tu, M. , Wang, D. , Liu, J. , Li, Y. , Li, Z. , Wang, Y. *et al* (2017b) CRISPR/Cas9‐mediated efficient targeted mutagenesis in grape in the first generation. Plant Biotechnol. J. 1–12. 10.1111/pbi.12832 PMC5866948 28905515

[pbi12884-bib-0045] Xie, K. , Minkenberg, B. and Yang, Y. (2015) Boosting CRISPR/Cas9 multiplex editing capability with the endogenous tRNA‐processing system. Proc. Natl Acad. Sci. 112, 3570–3575. 25733849 10.1073/pnas.1420294112 PMC4371917

[pbi12884-bib-0046] Yuan, Y. (2011) Research progress of kiwifruit in tissue culture In 2011 International Conference on Future Computer Science and Application (FCSA 2011), Vol. 4, pp. 17–20.

