# Supplementary material for: Optimized paired‐sgRNA/Cas9 cloning and expression cassette triggers high‐efficiency multiplex genome editing in kiwifruit
Source: Plant Biotechnol J. 2018 Feb 6;16(8):1424–33. doi: 10.1111/pbi.12884 (PMC6041439; doi:10.1111/pbi.12884)
Supplement: Supplementary file 1 — Figure S1 Overall structures of vectors used in the CRISPR/Cas or PTG/Cas9 systems. Figure S2 PCR validation and Sanger sequencing of PCR products amplified from paired‐sgRNA/Cas9 vectors. Figure S3 Validation of transgene‐positive cells in G418‐resistant callus lines using Cas9‐specific primers SP‐DL/SP‐R. Figure S4 Relative expression of sgRNAs in kiwifruit. Table S1 Primers used for vectors construction, mutation detection, off‐target analysis and qPCR. Table S2 Summary of mutagenesis frequencies in positive clones selected. [file PBI-16-1424-s001.pdf]

Table S1. Primers used for vectors construction, mutation detection, off-target analysis and qPCR.

Primer name	Sequence (5'-3')
F1	GTGTGGTGTTGTCTGCTCTATGTTT
R1	CCAGCGCCCAATCATTTACCCTC
F2	AGTTGTTTGAATACTATGACCAGGATCTCCC
R2	GCAGCATATACGCGGAAAGAATTATAAACGAT
crispr-gRNA1-F	GGTCTCTgattCAGGTCTGTCCCATCAAGATGTTTTAGAGCTAGAAATAG
crispr-gRNA2-R	GGTCTCTAAACCTAAGCCAGTATCAGACTCCAATCACTACTTCGTCTCTA
crispr-gRNA3-F	GGTCTCTgattACTCGATGGATGATGATATAGTTTTAGAGCTAGAAATAG
crispr-gRNA4-R	GGTCTCTAAACGTCTTTAACAGTTAAACCATAATCACTACTTCGTCTCTA
ptg-gRNA1-F	GGTCTCTTGCACAGGTCTGTCCCATCAAGATGTTTCAGAGCTATGCTGGA
ptg-gRNA2-R	GGTCTCTAAACCTAAGCCAGTATCAGACTCCTGCACCAGCCGGGAATCGA
ptg-gRNA3-F	GGTCTCTTGCAACTCGATGGATGATGATATAGTTTCAGAGCTATGCTGGA
ptg-gRNA4-R	GGTCTCTAAACGTCTTTAACAGTTAAACCATTGCACCAGCCGGGAATCGA
SP-DL	GTCGTGCTCCACATGTTGACCGG
SP-R	CCCGACATAGATGCAATAACTTC
GF	aGAGACCGGTCTCGGTTTCAGAGCTATGCTGGAAACAGC
GR	AGCTCGAGAGGCGCGAAAAAAGCACCGACTCGGTGCCACTTTTTCAAGTTGAT
U6-1-F	GACCGGTAAGGCGCGAGAAATCTCAAAATTCCGGCAGAACAA
U6-1-C	CGAGACCGGTCTCtAATCACTACTTCGTCTCTAACCATATAT
U6-1-R	CACTGGTGCTTTGTTAATCACTACTTCGTCTCTAACCATATAT
TF	AACAAAGCACCAGTGGTCTAGTGGTAGAATAGTACCCTGCCACGGTACAGACCCGGGT
TR	CGAGACCGGTCTCtTGCACCAGCCGGGAATCGAACCCGGGTCTGTACCGTGG
gRNA-R	GCACCGACTCGGTGCCAC
gRNA1-F	CAGGTCTGTCCCATCAAGATGTTTCAGAGC
gRNA2-F	GGAGTCTGATACTGGCTTAGGTTTCAGAGC
gRNA3-F	ACTCGATGGATGATGATATAGTTTCAGAGC
gRNA4-F	ATGGTTTAACTGTTAAAGACGTTTCAGAGC
Achn107181-F	TGAGAGATTCCGTTGCCCAGAAGT
Achn107181-R	TTCCTTACTCATGCGGTCTGCGAT
PP2A-F	GCAGCACATAATTCCACAGG
PP2A-R	TTTCTGAGCCCATAACAGGAG
T1-F	GTGTGGTGTTGTCTGCTCTATGTTT
T1-R	CTCTCTCACATTGGTGGGGTTGT
T2-F	ACAACCCCACCAATGTGAGAGAG
T2-R	CCAGCGCCCAATCATTTACCCTC
T3-F	AGTTGTTTGAATACTATGACCAGGATCTCCC
T3-R	CATATGGCTGTCCACCTAGCATTGC
T4-F	AGCAATGCTAGGTGGACAGCCATATG
T4-R	GCAGCATATACGCGGAAAGAATTATAAACGAT
OT1-F	TCTCCACCTTCTTTATGC
OT1-R	TGCTATTTGTCCGTCTC
OT2-F	ATAACCAAGGCAGAGC
OT2-R	AACCCATGACCACTAAC
OT3-F	TGGCAATGAACACCTC
OT3-R	AACCCATGACCACTAACT
OT4-F	TCACCAGGTTCAAAGC
OT4-R	GTTAGCGTCCCTCACA

Table S2. Summary of mutagenesis frequencies in positive clones selected.

Vector ID	sgRNA	No. of clones analyzed	No. of mutated clones	Mutation frequency (%)
A1	sgRNA1	120	9	7.50%
	sgRNA2		9	7.50%
	sgRNA1&sgRNA2		9	7.50%
A2	sgRNA3	280	0	0.00%
	sgRNA4		19	6.78%
	sgRNA3&sgRNA4		0	0.00%
B1	sgRNA1	120	109	90.83%
	sgRNA2		98	81.67%
	sgRNA1&sgRNA2		98	81.67%
B2	sgRNA3	260	190	73.07%
	sgRNA4		158	65.38%
	sgRNA3&sgRNA4		156	60.00%

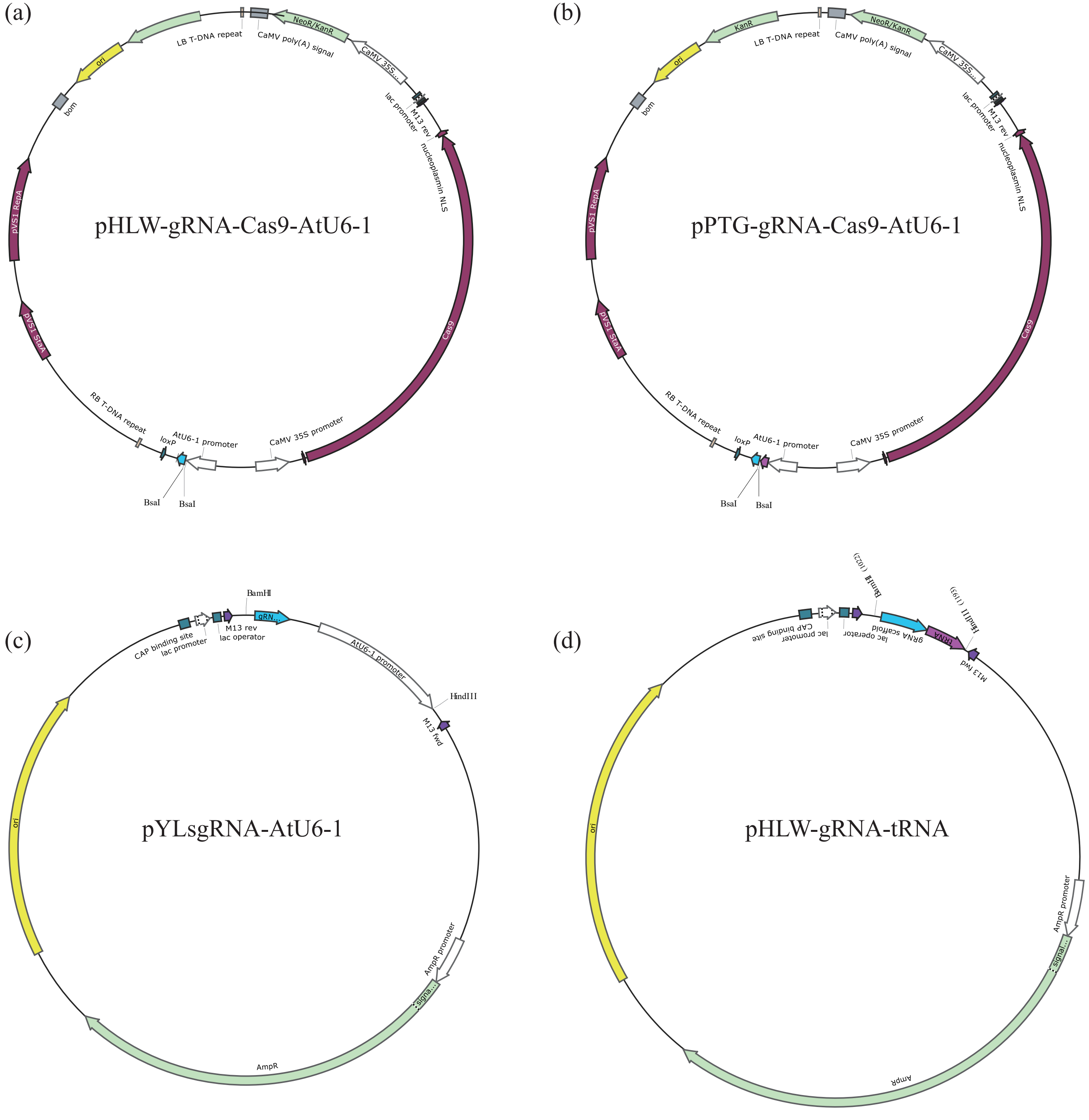

Figure S1. Overall structure of vectors for CRISPR/Cas system. (a) Overall structure of the Cas9 binary expression vector for CRISPR/Cas9 system. (b) Overall structure of the Cas9 binary expression vector for PTG/Cas9 system. (c) Overall structure of the sgRNA intermediate vector for CRISPR/Cas9 system. (d) Overall structure of the sgRNA intermediate vector for PTG/Cas9 system.

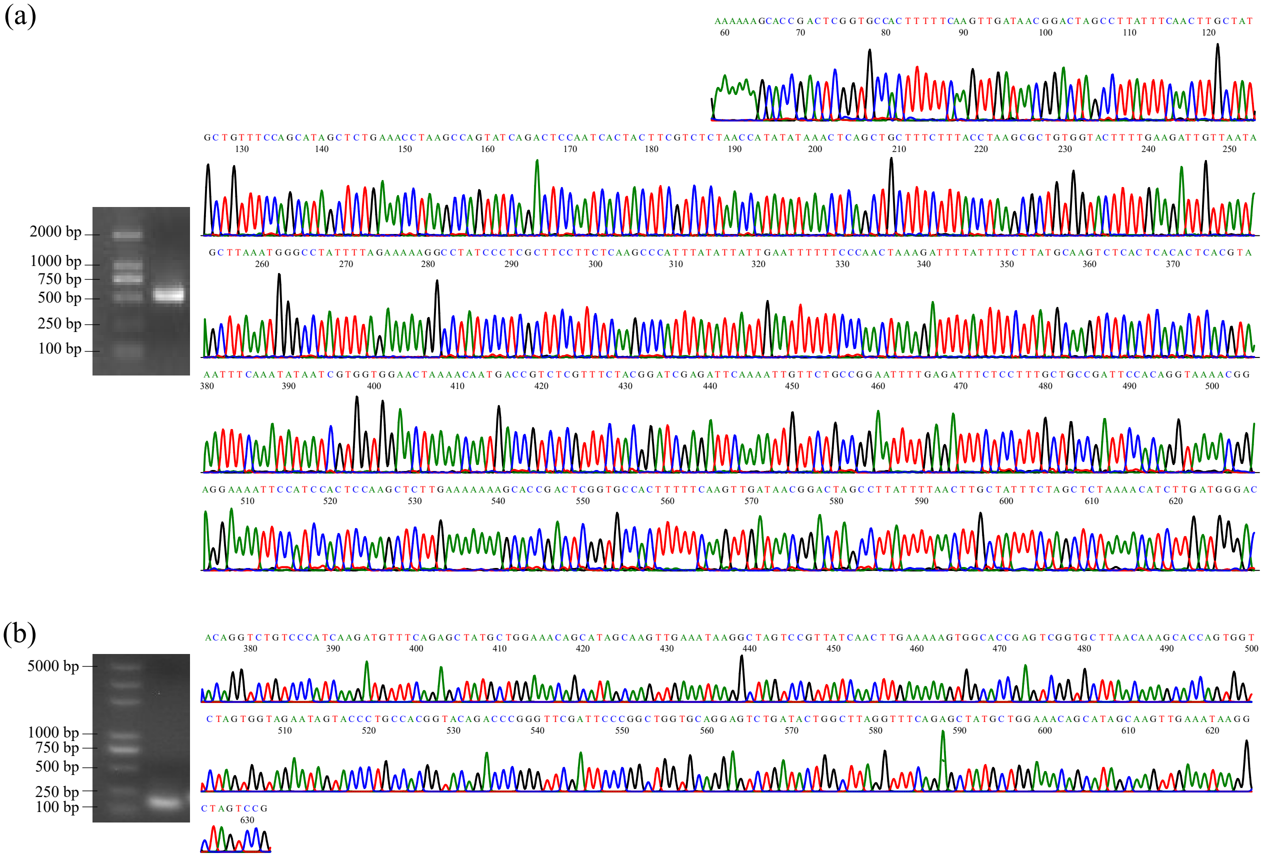

Figure S2. PCR validation and Sanger sequencing of PCR products amplified from paired-sgRNAs/Cas9 vectors. (a) Validation for CRISPR/Cas9 system. (b) Validation for PTG/Cas9 system.

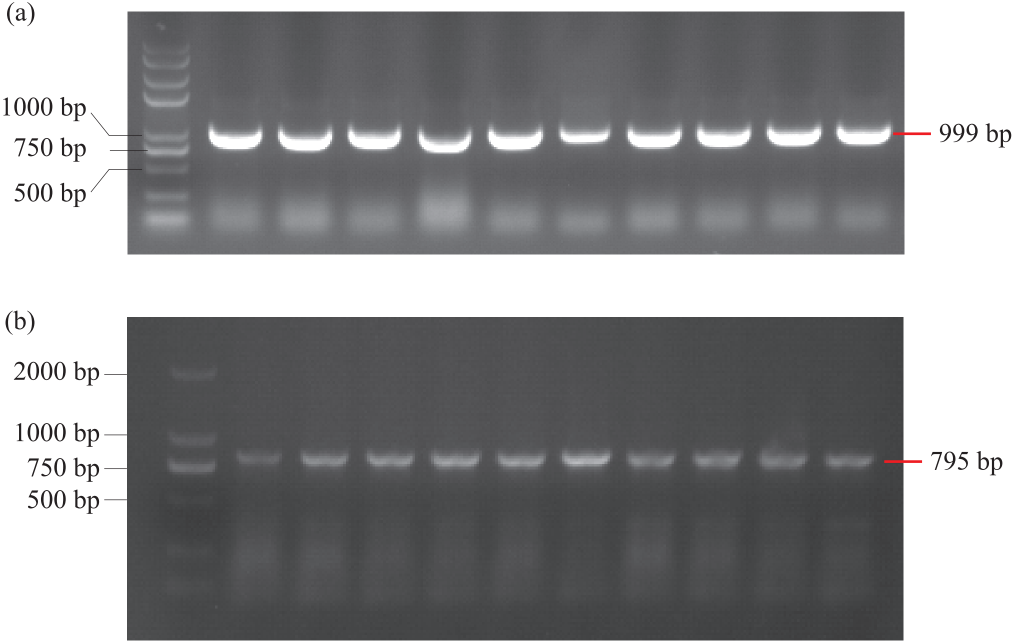

Figure S3. Transgenic positivity check in G418-resistance callus lines with Cas9 specific primers SP-DL/SP-R. (a) Representation of CRISPR/Cas9 system. (b) Representation of PTG/Cas9 system.

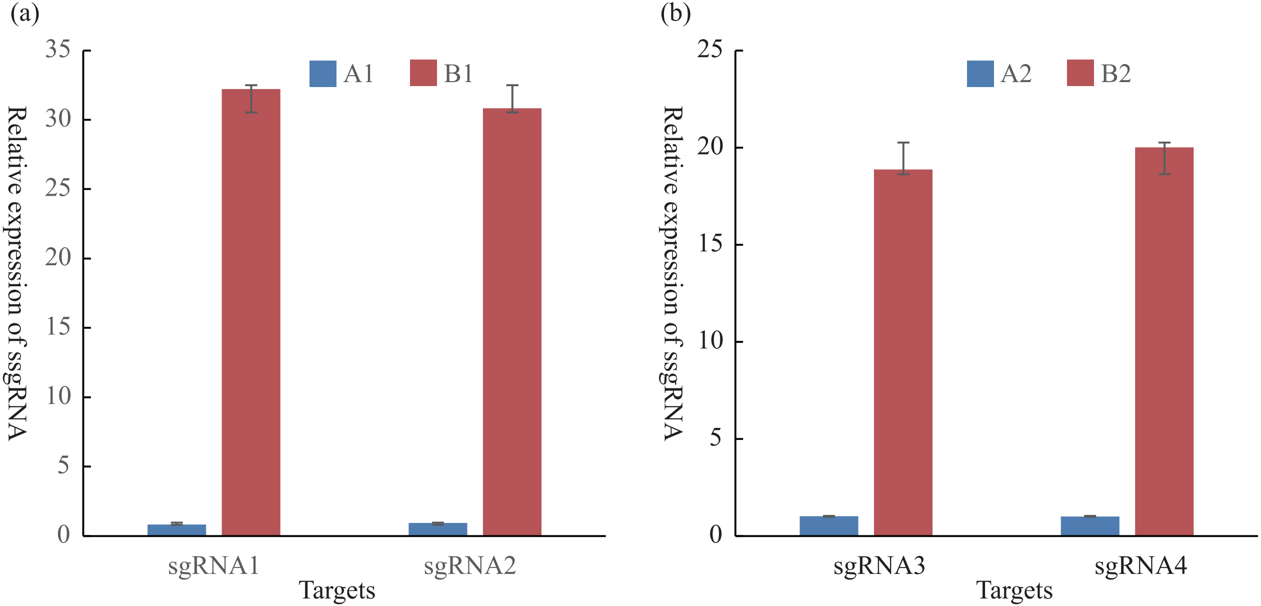

Figure S4. The relative expression of ssgRNAs. (a) The relative expression levels of sgRNA1 and sgRNA2 in kiwifruit G418-resistance callus lines transformed with A1/B1. (b) The relative expression levels sgRNA3 and sgRNA4 in kiwifruit G418-resistance callus lines transformed with A2/B2.
